# Potential of low-density genotype imputation for cost-efficient genomic selection for resistance to *Flavobacterium columnare* in rainbow trout (*Oncorhynchus mykiss*)

**DOI:** 10.1186/s12711-023-00832-z

**Published:** 2023-08-14

**Authors:** Clémence Fraslin, Diego Robledo, Antti Kause, Ross D. Houston

**Affiliations:** 1 The Roslin Institute and Royal (Dick) School of Veterinary Studies, University of Edinburgh, Easter Bush, Midlothian, EH25 9RG UK; 2 Natural Resources Institute Finland (Luke), Myllytie 1, 31600 Jokioinen, Finland; 3 Benchmark Genetics, Edinburgh Technopole, 1 Pioneer Building, Penicuik, EH26 0GB UK

## Abstract

**Background:**

*Flavobacterium columnare* is the pathogen agent of columnaris disease, a major emerging disease that affects rainbow trout aquaculture. Selective breeding using genomic selection has potential to achieve cumulative improvement of the host resistance. However, genomic selection is expensive partly because of the cost of genotyping large numbers of animals using high-density single nucleotide polymorphism (SNP) arrays. The objective of this study was to assess the efficiency of genomic selection for resistance to *F. columnare* using in silico low-density (LD) panels combined with imputation. After a natural outbreak of columnaris disease, 2874 challenged fish and 469 fish from the parental generation (n = 81 parents) were genotyped with 27,907 SNPs. The efficiency of genomic prediction using LD panels was assessed for 10 panels of different densities, which were created in silico using two sampling methods, random and equally spaced. All LD panels were also imputed to the full 28K HD panel using the parental generation as the reference population, and genomic predictions were re-evaluated. The potential of prioritizing SNPs that are associated with resistance to *F. columnare* was also tested for the six lower-density panels*.*

**Results:**

The accuracies of both imputation and genomic predictions were similar with random and equally-spaced sampling of SNPs. Using LD panels of at least 3000 SNPs or lower-density panels (as low as 300 SNPs) combined with imputation resulted in accuracies that were comparable to those of the 28K HD panel and were 11% higher than the pedigree-based predictions.

**Conclusions:**

Compared to using the commercial HD panel, LD panels combined with imputation may provide a more affordable approach to genomic prediction of breeding values, which supports a more widespread adoption of genomic selection in aquaculture breeding programmes.

**Supplementary Information:**

The online version contains supplementary material available at 10.1186/s12711-023-00832-z.

## Background

Aquaculture production has increased substantially over the past decades and is now supplying more aquatic products than fisheries [1]. Compared to livestock production, the domestication of most aquaculture species is recent and not all species benefit from modern selective breeding programmes [2]. Nonetheless, selective breeding has been successfully implemented for a large number of aquaculture species, and the recent development of high-throughput genotyping technologies, such as single nucleotide polymorphism (SNP) arrays, has opened the gate for the implementation of genomic selection for the most important species [2–4]. Genomic selection uses genome-wide marker information (mainly SNPs), to generate genomic relationship matrices, to predict the breeding value of genotyped selection candidates based on genotype and phenotype information that is obtained on a reference population [5, 6]. In aquaculture breeding programmes, many traits under selection cannot be measured directly on the candidates (e.g. fillet yield or disease resistance traits), and thus are measured on their full and half-sibs [7]. These so-called sib traits are the perfect target for the implementation of genomic selection because it captures the within-family genetic variation in addition to the between-family genetic variation. Over the recent years, a large number of studies have demonstrated that the application of genomic selection significantly improves the response to selection in aquaculture breeding programmes [2, 8–11].

The late implementation of genomic selection in aquaculture breeding programmes compared to terrestrial livestock species is partly due to the lack of high-throughput genotyping platforms for most species and due to the significant cost of genotyping the large number of individuals required for efficient genomic selection. Therefore, to date, genomic selection has only been implemented for a handful of aquaculture species that have the largest production value, and typically by the largest companies. Several strategies have been investigated to reduce genotyping costs and make genomic selection more affordable for small- and medium-scale breeding programmes, such as genotyping only a proportion of the individuals [12, 13], pooling DNA to build a reference population [14–16], or using medium- and low-density (LD) SNP panels that are typically cheaper to produce.

To date, many studies have investigated the potential of using LD SNP panels for genomic selection in various aquaculture species and they concluded that LD panels containing between 1000 to 2000 SNPs [17, 18] and 6000 SNPs [19]. Depending on the species and the trait (reviewed in Song et al. [11]), such LD panels are sufficient to achieve an accuracy of genomic prediction similar to that obtained with a medium- or high-density (HD) panel. In those studies, further reduction of the density to hundreds of SNPs resulted in a significant drop in accuracy [17, 20]. This issue could potentially be resolved via the use of imputation. Imputation predicts the missing genotypes in a LD-genotyped target population using information from a HD-genotyped reference population. Imputation relies on linkage disequilibrium information in a population-based imputation approach, or on linkage information in a family-based imputation approach [21]. The usefulness of imputation in genomic prediction has been studied for various farmed crops and animals [22–25] and is now implemented on a routine basis in cattle genomic selection. A few recent studies have investigated the impact of imputing LD to medium- or HD genotypes on the accuracy of genomic prediction in several major aquaculture species such as Atlantic salmon, rainbow trout and tilapia [26–33]. These studies have shown that a cost-efficient genomic selection could be achieved with a combined approach of LD genotyping and imputation.

One example of a programme where the benefits of such cost-efficient genotyping approaches could be realised is the Finnish rainbow trout breeding programme. This programme was established in the late 1980s and relies on pedigree-based information obtained by the initial rearing of families in separate tanks until the fish are big enough to be tagged and pooled together in larger tanks [34, 35]. In recent years, the columnaris disease (CD) caused by *Flavobacterium columnare* has become a major concern for rainbow trout farming in Finland. *Flavobacterium columnare* is a bacterium distributed worldwide that affects fresh water fish under warm water conditions, usually when temperatures are above 18–20 °C, but it has also been reported to affect salmonids in cooler water conditions [36–39]. *F. columnare* causes acute and chronic infections with the main symptoms being tissue and gill necrosis especially in small fish, leading to high mortality if the disease is not treated [37, 39, 40]. In a recent study on resistance to *F. columnare* in two Finnish rainbow trout populations, genetic variation was observed and quantitative trait loci (QTL) associated with resistance to this disease were identified, thus the use of genomic selection (and/or marker assisted selection) was recommended to improve this trait [41, 42]. Implementing genomic selection may speed up genetic gain for various traits including resistance to CD, but to date the cost of genotyping remains prohibitive for the Finnish breeding programme. The aim of this study was to assess the efficiency of genomic selection to improve rainbow trout resistance to *F. columnare* using LD SNP panels that were built in silico combined with imputation using three SNP selection strategies: (i) randomly sampled SNPs along the chromosomes, (ii) equally-spaced SNPs on each chromosome and (iii) most significant SNPs based on a genome-wide association study (GWAS) results.

## Methods

### Fish rearing, disease outbreak management and genotyping

The fish used in this study were from the Finnish national breeding programme for rainbow trout, managed by the Natural Resources Institute Finland (LUKE). Fish rearing, phenotyping and genotyping have been described by Fraslin et al. [41]. Briefly, in May 2019, 81 rainbow trout breeding candidates [33 females (dams) and 48 males (sires)] were selected among 567 fish from the Finnish national breeding programme, based on their relationships and genetic contribution to maintain a predetermined inbreeding coefficient of less than 1% per generation. The 33 dams and 48 sires were mated to create 105 full-sib families with one dam mated to one to four sires (2.2 in average) and one sire mated to two to four dams (3 on average). The optimal genetic contributions method was used to select the parents with high selection index and low relationship, to determine the number of matings allowed for each parent, and to minimize the kinship level of the offspring by minimizing the relationship between mating pairs [34]. Fifty mL of eggs from each mating were pooled after fertilisation and incubated together.

In June 2019, about 30,000 fry were separated into three fingerling tanks, resulting in about 100 fish per family per tank, at a multiplier farm of Hanka-Taimen Oy (Finland) that uses water from a nearby stream with naturally-occurring CD outbreaks. From the time of arrival at this multiplier farm (considered as day 0 of the study, corresponding to 52–53 days post-hatching), fish mortality and any signs of disease were monitored twice a day. On day 11 of the experiment, fish in all three tanks started to show signs of CD (saddleback lesions), and seven dead or dying fish were sampled and sent to a veterinarian to confirm the CD diagnosis. The presence of the pathogen was confirmed by PCR. From day 20 to 24, a piece of tail from 510 fish per tank, which were randomly chosen among the dead or dying fish with clear CD signs (considered as susceptible), was sampled for later DNA extraction. At day 26, the three tanks were treated following the veterinarian guidelines against *F. columnare* with an approved treatment of salt, chloramine and medical feed until day 32. On the last day of the experiment, day 99, a piece of tail was collected, for later DNA extraction, on about 506 fish per tank, which were randomly sampled among the fish still alive at that time (considered as resistant). In total, 3057 challenged fish (1538 susceptible and 1519 resistant) and 570 fish from the parental generation (including the 81 parents) were genotyped using the 57K SNP Axiom™ Trout Genotyping Array [43]. The genotypes of all 3624 individuals were called together in a single run using the Axiom Analysis Suite software (v.4.0.3.3) with the recommended standard SNP quality controls. Only SNPs that were classified as “highly polymorphic” by the software were kept for further quality control (n = 36,020 SNPs, corresponding to 62.6% of the SNPs). The software Plink (v.1.9) [44] was used to perform quality control on SNPs and individuals based on deviation from the Hardy-Weinberg equilibrium (*p-value* ≤ 10^−6^, n = 5973 SNPs removed)*,* minor allele frequency (≥ 0.05, n = 445 SNPs removed), SNP call rate (≥ 0.95, n = 1942 SNPs removed), and individual call rate (≥ 0.9, n = 8 individuals removed). The final dataset comprised 2874 challenged fish and 469 fish from the parental generation (including 78 parents of the challenged fish), all genotyped for 27,907 SNPs. Those 28K SNPs were considered as the HD panel for the remaining of the analysis.

Parentage assignment was performed in two steps. First, a subset of 200 SNPs with a 100% call rate in both generations was used to recover the pedigree of the offspring with no missing parents using the APIS R package [45] with a mismatch assignment value set to 1%. Since APIS does not perform parentage assignment when one of the parents is missing, the genomic relationship matrix (GRM) built with the HD panel in the GCTA software [46] was used to infer the half-sib family when one parent was missing, recovering only the parent that was genotyped. The full pedigree was recovered for 96.6% of the fish, with only 88 fish having one parent unassigned/missing and 10 fish with no parents at all. The quality of the pedigree was then checked with the option “parentage_test” with an error rate threshold of 0.05 (“/ert mm 0.05”) from FImpute [47] using the HD genotype and all the fish in the dataset. No progeny-parent mismatches were detected and for the 179 fish with one or two missing parents, the software was unable to suggest a suitable parent when the option “find_match_cnflt” was used, so the pedigree was not modified.

### In silico low-density panels

The impact of decreasing SNP density on genomic prediction was tested with LD SNP panels created in silico using three sampling methods. In the first method, SNPs were sampled randomly on each chromosome, with the number of SNPs sampled from a given chromosome being proportional to its physical length in the *O. mykiss* reference genome (Omyk_0.1) [48]. This random selection method will be referred to as RandLD (random low-density). In the second sampling method, referred to as EquaLD (equally spaced low-density), SNPs were selected such that they were equally spaced on each chromosome. For these two methods, we used the CVrepGPAcalc package [26] to create 10 panels with densities of 300; 500; 700; 1000; 3000; 5000; 7000; 10,000; 15,000 and 20,000 SNPs. Replicates were allowed to overlap by chance and the final number of SNPs within each panel was allowed to vary slightly from the target density. On average, the RandLD panels created contained between 10 and 15 more SNPs than the target density (see Additional file 1: Table S1). All the replicates for each density contained the same number of SNPs. The EquaLD panels were more variable and contained, on average, between one and two less SNPs than the target density for LD panels from 300 to 1K and, on average, between 2 and 104 more SNPs for densities from 3 to 15K. For both methods, the 20K LD panel contained about 1K less SNPs than targeted, with 19,803 SNPs for the RandLD panels and on average 19,039 SNPs (± 55 SNPs) for the EquaLD panels (see Additional file 1: Table S1)

Finally, for the third SNP sampling method, we used the results of the GWAS for resistance to *F. columnare* to create top low-density (TopLD) panels based on the *p*-value estimated by the GWAS in order to investigate the effect of including SNPs with a significant effect on resistance into the LD panel. The SNP effect and *p*-value were computed in a GWAS performed with a mixed linear model association (mlma) with the leave-one-chromosome-out (loco) option implemented in the GCTA software [46] using the model presented in Fraslin et al. [41] with resistance being analysed as a binary trait (0 = alive, and 1 = dead) and the tank number included as a fixed effect. One of the pitfalls of the creation of those TopLD panels is due to the SNP effects being estimated in a GWAS that includes the whole population, thus validating those SNP effects on a sub-sampling of the population in a genomic prediction approach (validation group) can lead to inflated prediction accuracies. In order to avoid this issue and to estimate the SNP effects in a group that is independent from the validation set, we used the same “leave-one-group” out approach in a five-fold cross-validation scheme as defined for the evaluation of genomic prediction accuracy (see below) to perform 100 independent GWAS. Specifically, the fish were randomly separated into five groups, one of them (validation set representing 20% of the population) was excluded from the analysis and the phenotype and genotype information of the remaining 80% of the fish (training set) were used in the GWAS to estimate each SNP effect and *p*-value for association to resistance, and this was repeated for the five groups. This was replicated 20 times to match the 20 replicates of the genomic prediction evaluation, so in total 100 GWAS were performed. The SNPs were then ranked, within each GWAS, from the lowest *p*-value (most significant association) to the highest *p*-value (least significant association) and the N first SNPs were sampled to create a TopLD panel. One hundred TopLD panels were created for each density and we tested six densities representing the best 300, 500, 700, 1000, 3000 and 5000 SNPs. Since the SNPs were selected based on the GWAS results, not all the chromosomes were represented in the lower density TopLD panels, and chromosomes 3 and 5 were over-represented due to the presence of major QTL associated with resistance to *F. columnare* on these chromosomes [41, 42].

### Imputation of low-density panels to a high-density of 27,970 SNPs

Imputation was performed only for the RandLD and EquaLD panels using the FImpute3 software [47]. LD genotypes from the offspring were imputed back to the full ~ 28K SNPs using a combined population and pedigree-based imputation method with the HD-genotyped parents (n = 469 fish, including n = 78 parents) as the reference population. The “parentage_test” option was used with an error rate threshold of 0.05 (“/ert mm 0.05”) to find progeny-parent mismatches based on the pedigree, and in case of Mendelian inconsistency between progeny and parents for non-missing genotypes, the original genotypes were kept intact using the option “keep_og”. The accuracy of imputation was estimated as the Pearson correlation coefficient between true and imputed genotypes only for the SNPs that were removed to create the LD panels. After imputation, another quality control was performed and imputed SNPs with a MAF lower than 0.05 were removed.

### Genomic evaluation of low-density SNP panels before and after imputation

The (genomic) estimated breeding values [(G)EBV] of fish were computed using the following mixed linear best linear unbiased prediction (BLUP) animal model based on pedigree only (PBLUP) or genomic only (GBLUP) information using the BLUPF90 software [49]:

$$\mathbf{y}=\mathbf{X}\mathbf{b}+\mathbf{Z}\mathbf{a}+\mathbf{e},$$

where $\mathbf{y}$ is the vector of disease resistance phenotypes analysed as a binary trait (0 = alive; and 1 = dead), $\mathbf{b}$ is the vector of the fixed effect (rearing tank) with $\mathbf{X}$ the corresponding incidence matrix, $\mathbf{e}$ is the vector of residuals and $\mathbf{a}$ is the vector of random additive genetic effects with $\mathbf{Z}$ is the corresponding incidence matrix. The vector of random additive genetic effects followed a normal distribution $\mathbf{a}\sim \mathrm{ N}\left(0,\mathbf{A}{\upsigma }_{\mathrm{g}}^{2}\right)$ or $\mathbf{a}\sim \mathrm{ N}\left(0,\mathbf{G}{\upsigma }_{\mathrm{g}}^{2}\right)$ with ${\upsigma }_{\mathrm{g}}^{2}$ being the estimated genetic variance and $\mathbf{A}$ the pedigree-based relationship matrix used in the PBLUP analysis and $\mathbf{G}$ the genomic-based relationship matrix used in the GBLUP analysis. The efficiency of genomic prediction was estimated by a fivefold cross-validation procedure using the Monte-Carlo “leave-one-group-out” method. The phenotypes of 20% of the fish (validation set) were masked, and their (G)EBV were predicted using the phenotype and genotype information of the remaining 80% fish (training set). This procedure was repeated 20 times for the PBLUP, and the GBLUP with all 28K SNPs (HD-GBLUP) and for each of the 10 replicates of both the RandLD and EquaLD panels, pre- and post-imputation. For the TopLD panels, genomic prediction was only performed for the un-imputed panels, and since the groups created for the cross-validation procedure were the same as those used to select the SNPs in the panels, the performance of each TopLD panel was only tested within its corresponding validation set.

The performance of genomic prediction was assessed by estimating the accuracy of genomic prediction and the bias [50]. The accuracy was computed, for each SNP panel, as the mean over the 100 replicates of the correlation between the (G)EBV and the true phenotype of the fish in the validation group, divided by the square root of the genomic-based heritability (h^2^ = 0.21 as estimated in Fraslin et al. [41]). The bias was computed, for each SNP panel, as the regression coefficient of the true phenotype (on the y-axis) on the (G)EBV (on the x- axis). This coefficient is a measure of the degree of inflation and is expected to be equal to 1 in the absence of bias, a value below 1 represents an over-dispersion of (G)EBV and a value above 1 represents an under-dispersion of (G)EBV [51].

## Results

### Genomic prediction with the LD panels

Accuracies of the PBLUP and HD-GBLUP were previously reported in [41], i.e. the estimated pedigree-based prediction accuracy was 0.59 (± 0.080 sd) and the GBLUP genomic evaluation using the HD panel increased prediction accuracy by 14% (0.68 ± 0.076).

**Fig. 1:**
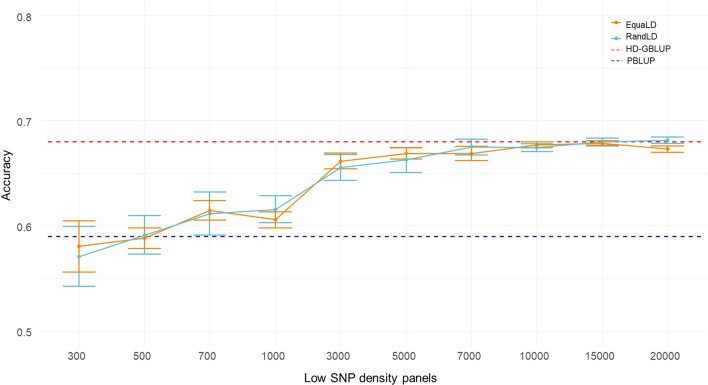
Accuracy of genomic prediction for resistance to *F. columnare* in rainbow trout, obtained with different low-density SNP panels (no imputation). The horizontal red dotted line is the average accuracy for the HD-GBLUP (28K) prediction (0.68), the horizontal blue dotted line is the average accuracy for the pedigree-based BLUP prediction (0.59). The light blue line is the accuracy obtained with the Random SNP sampling LD-panels (RandLD). The orange line is the accuracy obtained with the equally spaced SNP sampling LD-panels (EquaLD). The mean (dots) and standard deviations (bars) are taken from 10 replicates of each marker density

Decreasing the number of SNPs decreased the accuracy of genomic prediction (Fig. 1), and no significant difference was observed between the random or equally-spaced methods of SNP sampling. For both RandLD and EquaLD, prediction accuracies obtained with 300–500 SNPs were close to the accuracy obtained with the pedigree-based analysis. Encouragingly, prediction accuracies obtained with the LD panels including 7000 SNPs or more were close to the accuracy obtained with the HD panel (− 1% in accuracy compared with the HD-GBLUP). Accuracies obtained with 1000 SNPs were only 4% higher than those obtained with the pedigree-based analysis, whereas the accuracy obtained with only 3000 SNPs was 3% lower than the accuracies obtained with the HD panel and thus 11% higher than the accuracy obtained with the pedigree-based analysis only.

Variation among the 10 replicates was greater for lower densities with a bigger standard deviation (Fig. 1). With 300 SNPs, the highest accuracy obtained with one panel was 0.63 for RandDL and 0.61 for EquaLD, which was similar to the average accuracy obtained with 700 or 1000 SNPs. The lowest accuracy obtained with 300 SNPs was 0.53 for the RandLD panel and 0.55 for the EquaLD panel, which are significantly lower than the accuracy obtained with the pedigree-based analysis.

The accuracy and bias of genomic prediction obtained with the TopLD panels are in Table 1. For the lowest densities (300–1000), the accuracy of prediction obtained with SNPs selected based on their GWAS *p*-value (TopLD) was significantly higher than the accuracy obtained with panels of the same density when the SNPs were selected randomly or equally-spaced, except for EquaLD vs TopLD at 700 SNPs for which the difference was non-significant (*p*-value = 0.059, Wilcox test). For higher densities (3K and 5K), prioritising the SNPs based on the GWAS significantly decreased the accuracy of genomic prediction compared to RandLD or EquaLD panels of the same densities (*p*-value ranging from 0.002 to 0.05 for EquaLD 5K and RandLD 3K, respectively).

**Table 1:** Performance of the low-density SNP panels with the most significantly associated SNPs (TopLD strategy)

SNP density	Accuracy (mean ± sd)	Bias (mean ± sd)
300	0.62 ± 0.072	0.59 ± 0.078
500	0.66 ± 0.161	0.60 ± 0.143
700	0.63 ± 0.074	0.57 ± 0.079
1000	0.63 ± 0.074	0.56 ± 0.078
3000	0.64 ± 0.072	0.56 ± 0.074
5000	0.65 ± 0.074	0.57 ± 0.077

Values are the mean accuracy and mean bias obtained as average of the 100 replicates (5 groups * 20 replicates).

For all the TopLD panels, the GEBV obtained were highly biased with on average a bias of 0.575, which represents an over-dispersion of the breeding values. In contrast, the RandLD and EquaLD panels showed very little bias (see Additional file 1: Table S2).

### Imputation from low-density genotypes to 28K SNPs

**Fig. 2:**
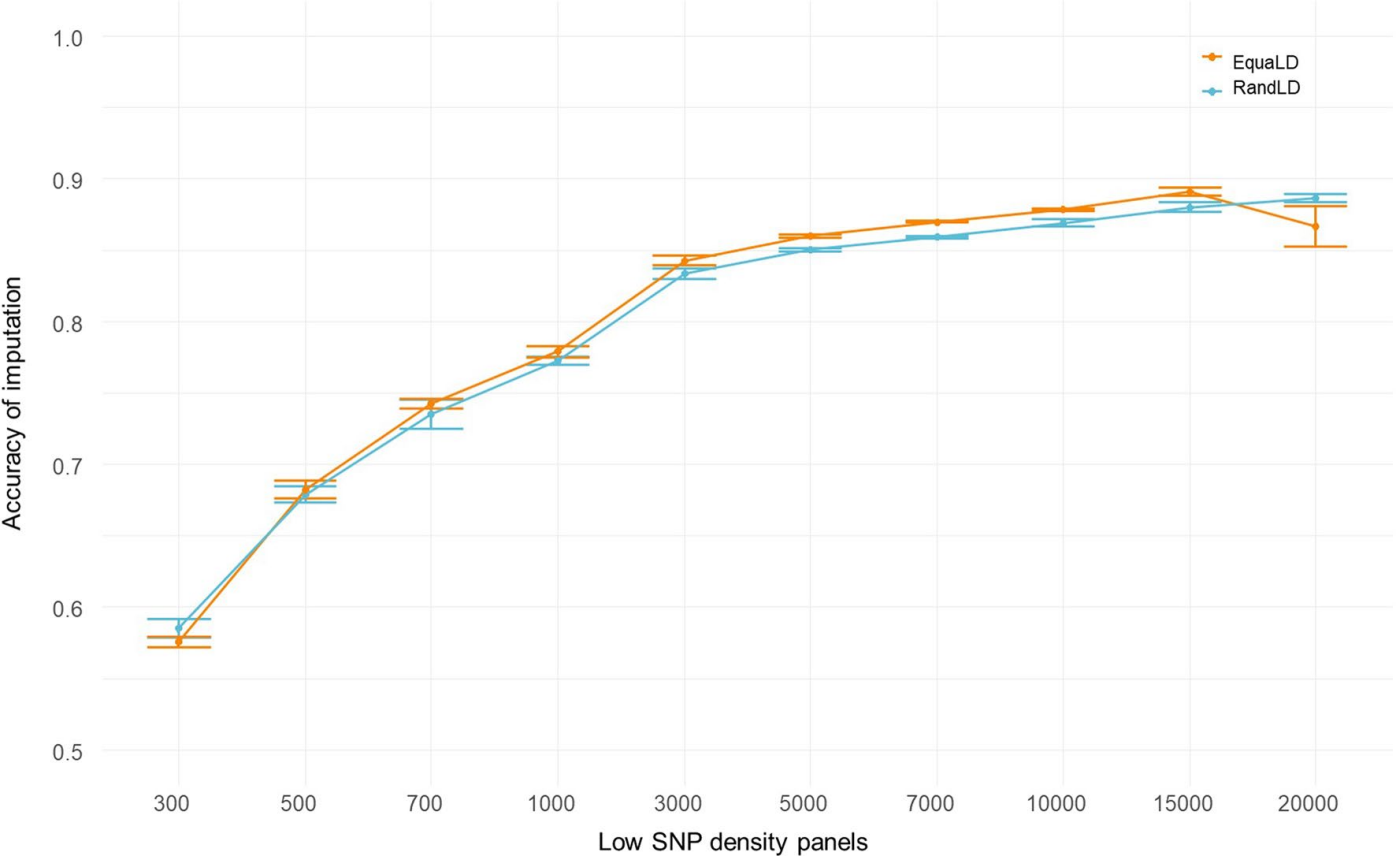
Imputation accuracy of LD-panels imputed to 28K SNPs. Accuracy measured as the Pearson coefficient between true and imputed genotypes for each individual and averaged over the 10 LD-panel replicates for each of the SNP densities. In blue results for the RandLD-panels (randomly sampled), and in orange for the EquaLD panels (equally spaced)

The imputation accuracies for both SNP selection methods are presented in Fig. 2. There was no significant difference in the accuracy of imputation for the two SNP selection methods (random vs. equally-spaced), except for the 20K SNP panels where the EquaLD panel had a lower imputation accuracy. Accuracy of imputation increased rapidly from 0.58 (± 0.004) for the 300-SNP EquaLD panel to 0.68 (± 0.005) for the 500-SNP EquaLD panel, and reached a plateau at around 0.86–0.89 from the 7000-SNP LD panels. The last important increase in imputation accuracy occurred between 1000 (0.77 ± 0.003) and 3000 SNPs (0.83 ± 0.003), and for higher densities imputation accuracy increased at a lower and steadier rate. There was a drop in the imputation accuracy at 20K SNPs for the EquaLD panel only but with a higher variability among panels (0.87 ± 0.014 sd).

The number of SNPs in each panel post-imputation was slightly smaller than in the HD panel due to the quality controls on the MAF being performed post-imputation. On average, 27K SNPs remained in the imputed panels for both SNP selection methods (min = 23,941 for 300 SNPs selected using the EquaLD equally-spaced sampling; max = 27,821 for the 20K SNPs RandLD randomly selected).

### Genomic prediction with the imputed LD panels

After imputation, for all starting SNP densities, the accuracy of genomic prediction for both SNP selection methods ranged from 0.63 to 0.65 with a plateau at 0.65 from the 3000-SNP density and above.

**Fig. 3:**
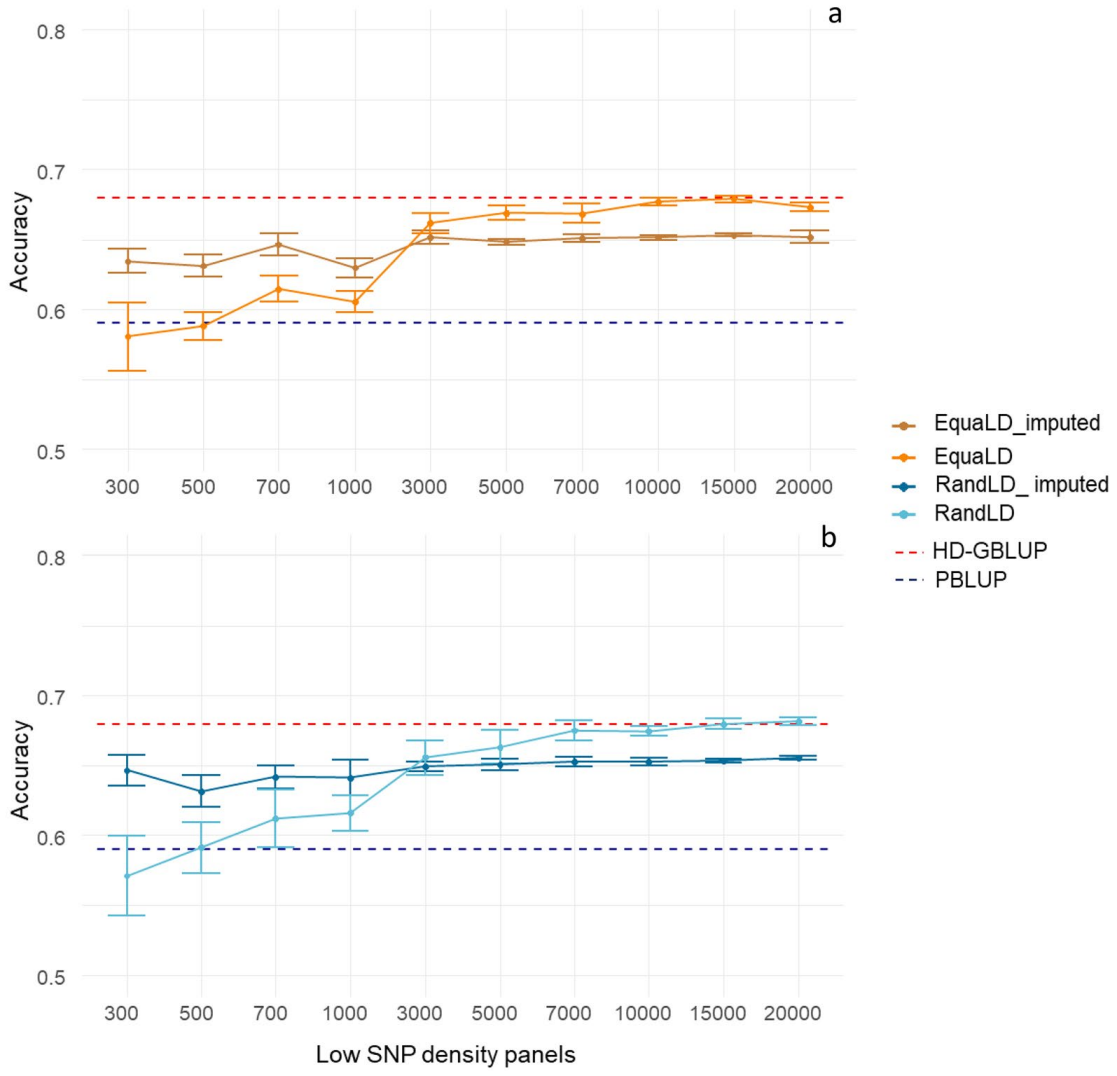
Accuracy of genomic prediction for resistance to *F. columnare* in rainbow trout, obtained with different SNP panels of different densities, before and after imputation, for (**a**) equally spaced SNPs and (**b**) randomly sampled SNPs. The horizontal red dotted line is the average accuracy for the HD-GBLUP (28K) prediction (0.68), the horizontal blue dotted line is the average accuracy for the pedigree-based BLUP prediction (0.59). **a** is for equally spaced SNP panels. The orange line is the accuracy value obtained with the equally spaced LD-panels (EquaLD) and the dark orange line is the accuracy obtained after imputation for those panels. **b** is for Random SNP panels. The light blue line is the accuracy value obtained with the Random LD-panels (RandLD) and the dark blue line is the accuracy value obtained after imputation for those panels

At the lowest densities (< 3000 SNPs, Fig. 3 and Table 1), imputation had a positive impact on the accuracy of genomic prediction, with accuracy values similar to those obtained with 3000 SNPs without imputation. The largest increase in accuracy of genomic prediction due to imputation was observed for the lowest density panel (300 SNPs), for which the accuracy of genomic prediction was increased by 11.6% for the RandLD (Table 2) and 7.5% for the EquaLD panels after imputation (see Additional file 1: Tables S2 and S3). For SNP densities of 500, 700 and 1000, imputation increased the accuracy of genomic prediction by 5% on average for the random sampling and by 5.5% on average for the equally-spaced sampling. Both sampling methods had similar performances after imputation.

**Table 2:** Accuracy of genomic prediction obtained for the random LD panels before and after imputation

SNP density	Accuracy of RandLD-panel	Accuracy of imputed RandLD-panel	Change in accuracy due to imputation (%)	Difference in accuracy between imputed LD panel and HD GBLUP (%)	Difference in accuracy between imputed LD panel and PBLUP (%)
300	0.57 ± 0.080	0.65 ± 0.078	+ 11.6	− 5.0	9.5
500	0.59 ± 0.077	0.63 ± 0.078	+ 6.3	− 7.1	6.9
700	0.61 ± 0.078	0.64 ± 0.077	+ 4.7	− 5.6	8.7
1000	0.62 ± 0.076	0.64 ± 0.076	+ 4.0	− 5.7	8.6
3000	0.66 ± 0.074	0.65 ± 0.075	− 1.0	− 4.5	10.0
5000	0.66 ± 0.075	0.65 ± 0.076	− 1.9	− 4.3	10.2
7000	0.68 ± 0.075	0.65 ± 0.076	− 3.4	− 4.0	10.5
10,000	0.67 ± 0.074	0.65 ± 0.075	− 3.4	− 4.0	10.5
15,000	0.68 ± 0.075	0.65 ± 0.076	− 4.1	− 4.0	10.6
20,000	0.68 ± 0.075	0.66 ± 0.077	− 4.0	− 3.6	11.0

RandLD = random sampling low-density panels, HD-GBLUP = high density panels (28K SNPs), PBLUP = pedigree-based BLUP

Mean of accuracy ± sd across all 100 values

Values for EquaLD are in Additional file 1: Tables S2 and S3

Surprisingly, the accuracy of genomic prediction obtained with 3000 SNPs was not significantly different before and after imputation, i.e. a small decrease of 1% for the random sampling and 1.5% for the equally-spaced sampling was observed. From the 5000-SNP density or higher, the accuracy of genomic prediction obtained after imputation was slightly lower than without imputation (Table 2) and (see Additional file 1: Tables S2 and S3), with on average a decrease of 3% compared to that obtained with the LD panels. After imputation, there was no or very little bias for all density panels with the average bias ranging from 1.00 to 1.01 (see Additional file 1: Table S2).

### Cost analysis

To assess the possibility of reducing genotyping costs by different genotyping and imputation practices, costs of genotyping and changes in accuracy were estimated for three SNP panels of decreasing densities (57K SNPs, 3K and 300 SNPs) for a breeding population of 8000 offspring and 200 parents, which reflects the Finnish breeding programmes of rainbow trout. The prices used are estimated for both LD panels since these are not currently available on the market. For the high-density SNP array (57K SNPs), genotyping costs approximately 20€ per sample when genotyping 8200 fish resulting in a total cost of ~ 164K €, which can be highly prohibitive for most breeding programmes. Under the assumption that a 3K SNP panel could be used to genotype all 8200 fish at a cost of 15€ per sample, this would represent a 25% reduction in genotyping cost for a 3% decrease in accuracy compared to the full HD panel. Another possible scenario is that all offspring are genotyped for a very low-density SNP panel (300 SNPs) at a cost of 7.5€ per sample, with parents genotyped for the existing 57K array at a higher cost of 30€ per sample (the price depends largely on the number of samples genotyped); in this scenario genotyping would cost 66K€. This reduces the genotyping cost by 60% compared to the price of the 57K SNP panel for only a 4% decrease in accuracy using the imputation approach. Moreover, *F. columnare* infects small fish, much before they can be individually tagged and identified. Therefore, even for an accurate pedigree-based evaluation, the offspring and parents need to be genotyped in order to recover the pedigree, meaning that the 300-SNP panel could be combined for both parentage assignment and imputation-based genomic selection.

## Discussion

In a previous work [41], we estimated a moderate heritability for resistance to *F. columnare* in this Finnish rainbow trout population (${h}_{\mathrm{g}}^{2}$ = 0.21) and we showed that genomic evaluation improved the accuracy of estimated breeding values compared to pedigree-based evaluation. The results obtained in the current study show that the use of low-density panels, combined with imputation, results in a higher accuracy of genomic prediction than pedigree-based PBLUP or using LD panels with no imputation, and could be an efficient way to implement genomic selection.

### Performance of the LD panels

In the current study, regardless of the SNP sampling method used to create the LD panels, we found that the use of 3000- to 7000-SNP panels without imputation would result in prediction accuracies comparable to those obtained with the full 28K HD panel. With a density of only 3000 SNPs, the prediction accuracy reached 96.4 to 97.3% of that of the HD panel, and with a density of 7000 SNPs prediction accuracies, which were equal to 98.3 and 99.3% of those the HD panel, were obtained with the RandLD and EquaLD panels, respectively. With the panels containing 300 or 500 SNPs, the accuracy was within the range of those obtained with PBLUP, with a non-significant decrease in accuracy by 3.3% or 1.6% for the 300-SNP panels for RandLD and EquaLD, respectively (see Additional file 1: Table S3). Those values are within the range of what has been reported in several other aquaculture species for various traits [17, 18, 20, 26, 29, 32, 52–55].

In our study, after the quality controls, the HD panel comprised 28K SNPs, which is considered as a medium-density panel in most animal species. This relatively small number of SNPs in the HD panel is due to the strict quality control and to the array being designed using SNPs discovered mainly in American rainbow trout populations, one Norwegian population and French double-haploid trout lines [43, 56] that probably differ from the Finnish population studied here. Indeed, most of the SNPs were filtered out in the Axiom Analysis step because of an absence of polymorphisms. Although relatively small, the number of SNPs in the 28K HD panel is within the range of previously reported densities, that range from 26 to 27K SNPs for the Chilean populations [28, 57] and from 29.8K to 34K SNPs for the French populations [58–61]. The number of SNPs required to accurately estimate breeding values in aquaculture species, and particularly in salmonids, is substantially smaller than those reported for terrestrial species that range from 49K SNPs for pig to 168K SNPs for Holstein cattle [62, 63]. The high accuracy of genomic prediction obtained with lower density panels in aquaculture species than in terrestrial species can be explained by the fact that predictions are obtained from close relatives (training population composed of full and half-sibs of the validation population) with very high within-family linkage disequilibrium as well as long-range linkage disequilibrium.

The high accuracy obtained with such low-density panels in aquaculture populations is most likely because aquaculture breeding programmes rely on large families and sib-testing, thus fish in the training and validation populations are closely related. In such populations, many individuals share long haplotype blocks since they have not been broken by recombination over generations, thus only a few SNPs per chromosome are needed to capture all the genomic information. Furthermore, in salmonids the limited male recombination across most of the genome [64, 65] is responsible for a slower decay of linkage disequilibrium and long un-recombined haplotype blocks being shared by individuals. In a previous study on Atlantic salmon, we showed that reducing both SNP density and the relationship level between training and validation populations led to a dramatic decrease in accuracy of genomic prediction [66]. The large family size in aquaculture breeding programmes can also explain the good performance of low-density panels, as previously reported in simulated sib-testing aquaculture breeding programmes [67–69] and in plants with similar breeding schemes [70]. The low impact of a decreased marker density on the within-family prediction accuracy due to large family size is explained by the fact that the GRM used in the prediction model are constructed within full-sib families and thus only a small number of markers is necessary to estimate relationships.

Another possible explanation is the existence of long-range linkage disequilibrium that has been previously characterised in salmonids species. In this rainbow trout population from LUKE, we estimated that the linkage disequilibrium between two SNPs separated by ~ 1 Mb is on average 0.11 (± 0.16) [41], i.e. lower than the 0.13 and 0.25 values estimated in other rainbow trout populations [52, 58]. In their study on rainbow trout resistance to BCWD, Vallejo et al. [52] showed that the accuracy of genomic prediction obtained with only 3K SNPs was almost as good as the accuracy obtained with 45K SNPs and partly explained these results by the high level of long-range linkage disequilibrium in this population (r^2^ ≥ 0.25 spanning over 1 Mb across the genome).

Finally, the optimal density panel to obtain near maximum accuracy without imputation varies slightly depending on the species, the size of the full-sib families and the architecture of the trait. However, for most aquaculture species and traits, a LD panel of 3000 SNPs was sufficient to reach an accuracy similar to the HD panel [9] with reference populations ranging from less than 600 [26, 29, 53, 55] to more than 2000 individuals [28, 71]. In Atlantic salmon, depending on the population and for a training size of ~ 600 fish, between 1 and 5K SNPs were required to reach the same accuracy as that obtained with 33K or 70K SNP panels [26, 29]. Yoshida et al. [28] showed that with only 3000 SNPs, the accuracy of prediction for resistance to *P. salmonis* in a population of 1938 rainbow trout was similar to the accuracy obtained with the HD panel of 27K SNPs using a Bayes C approach. Two *in* silico studies on five fish species (common carp, turbot, sea bass, rainbow trout and Atlantic salmon) [17, 18] compared LD panels to HD panels with a density ranging between 12 to 40K and found that LD panels containing between 3000 and 10,000 SNPs are sufficient to obtain near maximum accuracy. Recently similar prediction accuracies with 2000 SNPs and 4500 SNPs were obtained in flat oyster [20]. For European sea bass and sea bream populations, which were initially genotyped with about 60K SNPs, the use of a panel with only 6000 SNPs achieved accuracies that reached 90% of the accuracy of the HD panel [19]. In our study, as in most previously published studies [17–19, 55], further reduction of the density of the LD panels, i.e. between 700 and 1000 SNPs, resulted in a significant drop in the accuracy of genomic prediction but they remained higher than the accuracy obtained with PBLUP. In the case of LD panels with less than 1K SNPs, the GEBV were also more biased [52, 55] whereas in the current study the RandLD and EquaLD panels resulted in very little bias. This difference in the performance of the LD panels might also be due to the architecture of the trait studied, with potentially more markers required for polygenic traits with low heritabilities, and lower density panels performing better for more heritable traits with sizeable QTL, as simulated by Dufflocq et al. [30] and Dagnachew and Meuwissen [67]. In rainbow trout, Al-Tobasei et al. [55] reported that for fillet firmness that has a moderate to high heritability of 0.38, a LD panel composed of about 1K SNPs would have a similar predictive ability to that of the HD panel containing 50K SNPs. However, for fillet yield that has a lower heritability (0.20), more SNPs (11K) were required to reach a similar prediction accuracy.

Our results confirm that for rainbow trout, accurate genomic prediction can be achieved with a low marker density ranging from 3000 to 7000. The long-range linkage disequilibrium and low recombination rate that exist in salmonids and other aquaculture species along with the structure of the breeding programmes that rely on large families and close relationships between the training and validation populations are likely the main drivers for the good performance of the LD panels [66].

### LD-panels based on GWAS results

Previously, Fraslin et al. [41] detected a major QTL for resistance to *F. columnare* in this rainbow trout population, which increased the accuracy of genomic prediction when it was included in a GBLUP approach in which SNPs were weighted by their allele substitution effect. In the current study, we wanted to test the effect of including SNPs that are significantly associated with resistance to *F. columnare* in the LD panels (TopLD panels). This strategy significantly increased the accuracy of genomic prediction compared to the RandLD and EquaLD panels for densities of 1000 SNPs and lower. This was expected as the TopLD panels included the SNPs that were the most significantly associated with resistance, and thus the highest effect. Similarly, Al-Tobasei et al. [55] reported a higher accuracy for genomic prediction of fillet firmness in rainbow trout when using LD panels down to 800 SNPs that were prioritised based on the proportion of genetic variance explained, but with highly inflated predictions. However, in that study the GWAS was performed on the full population including the validation set for the genetic evaluation, which has an important impact on the bias of the predictions. Two studies on rainbow trout resistance to *F. psychrophilum* [52, 72] used LD panels of 70 or 49 SNPs that are located within previously detected QTL associated to this trait in a previous generation of the same population [73], and showed that these performed as well or even better than HD panels in terms of accuracy, and therefore could be used to accurately predict GEBV in subsequent generations. Those panels performed better than the LD panel without the major QTL [52], which highlights the importance of including SNPs that are associated with the trait of interest.

In the current study, for densities of 3000 and 5000 SNPs, RandLD or EquaLD performed significantly better than TopLD to predict the value of the fish in the validation set. In our population, the genetic architecture of resistance to *Flavobacterium columnare* was oligogenic, with the largest QTL on trout chromosome Omy3 and several minor QTL and a polygenic effect [41]. As the SNP density increased, more SNPs that are associated with QTL of smaller size were included in the panels, but SNPs from the main QTL were overrepresented, with a clear oversampling of SNPs from the chromosome Omy5. In a previous work by Calboli et al. [42] on two rainbow trout populations, we showed that there is a smaller QTL on Omy5 that spans 55 Mb with a large number of SNPs in very high linkage (r^2^ = 0.77 on average). This high linkage is responsible for a relatively strong effect of all the SNPs in this 55-Mb region and, as the density of the TopLD panels increases, more SNPs on chromosome Omy5 with redundant information are sampled, which do not contribute to the accuracy of genomic prediction since no or very little new information is added. The overrepresentation of SNPs from QTL in the TopLD panels led to highly biased prediction (on average 0.55). Furthermore, creating those LD panels based on GWAS results would not be applicable in practice. Indeed, not only does this analysis require that the GWAS be performed on the training population, but also it requires the development of a new LD panel for each population (and trait) since the QTL might not be shared between populations (and traits). For resistance to CD, it has been reported that some QTL are shared between two close Finnish populations [42] but not between the Finnish and American populations [41, 74]. The limited use of such LD panels would potentially increase their cost, and therefore defeat their use.

### Performance of imputed LD panels for genomic evaluation

The accuracy of imputation increased rapidly as the number of SNPs in the LD panel increased and from a 3000-SNP density upwards, the imputation accuracy ranged from 0.84 to 0.86 (± 0.001 − 0.003) for RandLD and EquaLD, and remained below 0.90 even when 20,000 SNPs were included in the LD panel (i.e. only about 8000 missing SNPs to impute). Those values are within the range of those previously reported for Atlantic salmon by Tsai et al. [29], and lower than those achieved by Kijas et al. [75] or Yoshida et al. [27] who used larger reference populations for imputation. The higher imputation accuracy obtained in other populations or in cattle could be due to their deeper pedigree, which improves phasing and therefore imputation.

Interestingly, in our study, the accuracy of genomic prediction post-imputation for both RandLD and EquaLD panels was quite stable, regardless of the starting density before imputation. The accuracy of genomic prediction did not seem to be affected by lower imputation accuracies, as observed for the lowest densities (300–700 SNPs). In a simulation study on rainbow trout, Dufflocq et al. [30] also showed that there were no significant differences in the accuracy of genomic prediction obtained after imputation with imputation error rates of 10, 5 or 1%. In most studies published on aquaculture species, the accuracy of genomic prediction after imputation was similar or slightly lower than the accuracy obtained with HD panels. Interestingly, in our study, we never reached the accuracy of genomic predictions obtained with the HD panel, and for densities higher than 5000 SNPs, the accuracy obtained with the LD panel was significantly higher than that obtained with the same panel after imputation. A similar observation was reported by Vallejo et al. [72] in their study on rainbow trout resistance to *F. psychrophilum*. They imputed a LD panel of 7K SNPs to a high-density of 32K SNPs and reported a lower accuracy of genomic prediction after imputation. However, since the actual genotyping was performed with 7K SNPs, the accuracy of imputation could not be estimated and this decrease could not be linked to imputation errors.

In order to better understand what could cause this decrease in accuracy post-imputation for LD panels with densities higher than 5K, we first imputed the HD panel to get a SNP call rate of 100% (as done in previous studies) [53, 59] and re-estimated the accuracy of the imputed HD panel. With the imputed HD panel, we obtained an accuracy of genomic prediction of 0.65 (± 0.077), which is lower than that of the HD-GBLUP prior to imputation (0.68 ± 0.076) but not significantly different from the accuracy of the imputed LD panels. We also performed a second test by setting all the genotypes that were missing in the un-imputed HD panel to missing in the imputed LD panel and re-estimating the accuracy of genomic prediction (see Additional file 2: Fig. S1). This resulted in a significant increase in the accuracy of genomic prediction compared to that obtained with the imputed LD panel, although it remained slightly lower than the accuracy of the un-imputed LD panel (see Additional file 2: Fig. S1). These results point towards an important impact of the missing genotypes, which is erased by imputation. In this dataset, 9% of the SNPs that passed the quality controls had a missing rate that differed significantly between live and dead fish. Those missing genotypes might provide information that is lost during imputation and thus result in the lower accuracy observed after imputation or they might generate bias and inflate the accuracy that is corrected by the imputation.

### Selective breeding for resistance after a natural disease outbreak

In aquaculture, selection for improved resistance to a pathogen is usually performed through a controlled infectious challenge [2, 10, 76]. While the opportunistic use of disease outbreak data and samples can be an effective approach for the genetic improvement of disease resistance, these outbreaks are unpredictable and can result in incomplete exposure to infection thus making it difficult to accurately measure resistance, which frequently results in underestimated heritability [77]. Moreover, with field data there is a risk of low predictability of resistance from one generation to another if the traits used to measure resistance are different, and this can be due to different infectious pathogen strains triggering different resistance mechanisms or to an imperfect diagnosis of resistance for surviving fish that were treated during the outbreak.

Bishop and Woolliams [77] introduced concepts for the genetic interpretation of disease resistance from field data and concluded that imperfect diagnostic or low prevalence results in underestimated heritabilities, and they observed a significant linear relationship between prevalence and heritability estimates for Atlantic salmon infected by infectious pancreatic necrosis virus (IPNv), both at the observed and underlying scale. In the current study, we do not know the real prevalence of the disease since the fish were treated against the pathogen to comply with the ethical law implemented in Finland. The fish considered as susceptible in the study all died in the first few days after the outbreak and presented clear signs of CD, and thus should be truly susceptible. The fish considered as resistant in the current study may have been alive at the end of the challenge because they were treated against the disease, which would affect the estimation of the resistance. However, due to the rapid mortality observed at the beginning of the outbreak and the relatively high density of fish in each tank, it is unlikely that some fish were never in contact with the pathogen and thus we can consider that all fish were indeed infected. Moreover, the co-localisation of QTL associated with resistance to *F. columnaris* detected in our previous study [41] and with resistance to *F. psychrophilum* [78], which are two closely-related [79] bacteria from the same genus, as well as the concordance with heritabilities estimated in a previous study using experimental infection challenges [74, 80, 81], suggest that the resistance trait measured in the current study is an accurate estimation of genetic resistance.

In our previous report on the same dataset [41], we discussed that, although natural field outbreaks are not ideal to study disease resistance from an academic point of view, they produce valuable production-relevant phenotypes and are usually also cheaper than experimental challenges. Indeed, experimental challenges require specific facilities, permissions and extensive knowledge of the pathogen, which are frequently cost prohibitive for small- or medium-scale breeding programmes. Furthermore, infectious challenges are usually performed using injection or immersion methods, which induce a stress factor, as reviewed by Fraslin et al. [76]. Mucus and skin represent very important physical and immune barriers against pathogens, which play an important role in fish resistance that is bypassed by injection challenges. As a result, the resistance mechanisms triggered by natural infection might differ from the mechanisms triggered by an infectious challenge as shown by Fraslin et al. [60, 78] who reported the detection of different QTL associated with resistance to *F. psychrophilum* in rainbow trout in an injection challenge, an immersion challenge and a natural outbreak in a farm. The genetic correlation between resistance to an experimental challenge and resistance under farm conditions has been evaluated in a small number of studies. High correlations have been reported in Atlantic salmon for resistance to *A. salmonicida* [82], *L. salmonis* [83] and for resistance to IPNv [84, 85] a disease for which a major QTL has been detected [86]. In rainbow trout, Wiens et al. [87] showed that three generations of selection for resistance to *F. psychrophilum* using an injection challenge increased the resistance after a natural outbreak. However, more recently, two studies on resistance to the amoebic gill disease (AGD) in Atlantic salmon [88, 89] estimated correlations close to 0 between resistance measured after an immersion challenge and resistance measure after a natural outbreak in the field. Both studies concluded that resistance measured after the immersion challenge was a poor predictor of resistance to AGD under farm conditions and that this experimental challenge should not replace a field test in the selective programme. The question of the validity of experimental challenges to select for resistance in the field still remains and selection for improved resistance using natural outbreaks, although imperfect, might still be the best option to increase disease resistance in aquaculture populations.

### Cost efficiency of genotyping strategies

In this study, we showed that using a low-density panel to genotype rainbow trout and perform a genomic prediction of resistance to CD would result only in a small reduction of the accuracy of prediction (3%) compared to the use of a high-density panel for a considerable reduction in cost (about 25%). However, the price of 15€ per sample for genotyping using a 3K SNP panel is hypothetical as such panels do not exist for rainbow trout, and in reality they could be more expensive than estimated, thus reducing the interest of low-density genotyping. One solution would be to incorporate those 3K SNPs on a multispecies SNP panel that would be produced in larger numbers and thus be less expensive. Such multi-species panels have been developed for various aquaculture species (*Sparus aurata* and *Dicentrarchus labrax* ([90], *Crassostrea gigas* and *Ostrea edulis* [91], or *Colossoma macropomum* and *Piaractus mesopotamicus* [92]). An interesting solution would be to develop a very low-density SNP panel or use targeted-genotyping-by-sequencing to genotype 300–500 SNPs (to account for a decrease in the number of SNPs post quality control) and combine it with imputation using high-density-genotyped relatives as reference population. Such very low-density panels could be developed to be specific to a population, could include SNPs located within QTL that are associated with traits of interest, and be used not only for genomic selection but also for parentage assignment. In breeding programmes, the number of traits in the selection index is quite large and including QTL that are associated with all of them would not be possible in very low-density panels. However, most of the traits are polygenic and there are very few traits of interest that are controlled by a major QTL [76]. The careful design of very low-density panels (equally-spaced SNPs along all the chromosomes) will maximise imputation accuracy and create affordable LD panels that would be highly efficient for genomic selection when combined with imputation. In aquaculture breeding programmes, to keep track of pedigree, either all full-sibs are reared together in family tanks until they are big enough to be individually tagged, and then families are mixed [93], or all fish from different families are pooled early in life in a common environment with the need to genotype the fish and perform parentage assignment [94]. In the case of breeding for resistance to *F. columnare*, outbreaks occur in very small fish, much before they can be individually tagged for identification. As a consequence, parentage assignment is necessary to recover the pedigree and is usually performed using a very low-density panel. The development of a new low-density panel with a slightly higher density would enable genomic selection as well as the necessary parentage assignment, and would only represent a small extra-cost to the standard approach.

In the current study, we analysed the relevance of using low-density panels for genomic selection as a way to reduce the genotyping cost with marginal loss in accuracy, which would allow low- and medium scale aquaculture breeding programmes to implement genomic selection. The use of low-density panels is also interesting for larger companies because for the same genotyping budget, it would allow to genotype more individuals. These additional genotyped individuals could be used to increase the training population size, which in turn could increase the accuracy of prediction [53, 67, 68], but this comes at the cost of phenotyping more individuals. Additional genotypes could also allow the genotyping of more candidates, and thus increase the selection pressure, which is also an important component of genetic gain.

## Conclusions

In conclusion, the use of low-density SNP panels may reduce the costs of genomic selection in rainbow trout without a major reduction in the prediction accuracy of breeding values. Using low-density SNP panels (about 3000 SNPs) or very low-density SNP panels (about 300 SNPs) combined with imputation using HD-genotyped parents would result in a decrease of prediction accuracy of only 3–4% compared to a HD-genotyped population, which corresponds to an increase of 10.5–11% compared to a pedigree-based prediction. The good performance of such low-density panels might be potentially valid for most aquaculture species with long-range linkage disequilibrium, low recombination rates and breeding programmes that rely on sib-testing with large family size. Our findings suggest that a cost-effective genomic evaluation to improve the accuracy of selective breeding in rainbow trout is feasible and low-density genotyping combined with imputation could be a way to speed-up the implementation of genomic selection in low- or medium-scale breeding programmes.

### Supplementary Information

**Additional file 1: Table S1.** Number of SNPs in the low-density panels. RandLD = random sampling of SNPs in the low-density panels. EquiLD = equidistant sampling of SNPs in the low-density panels. **Table S2.** Values of accuracy and bias of genomic prediction obtained for the random, equidistant and top LD-Panels before and after imputation. RandLD = random sampling of SNPs in the low-density panels. EquiLD = equidistant sampling of SNPs in the low-density panels. TOP-LD = top SNP low-density panels. Mean of accuracy ± sd across all 100 values. **Table S3.** Proportion of increase or decrease of the accuracy of genomic prediction obtained for the random, equidistant and top LD-panels before and after imputation compared to pedigree and HD-panels. RandLD = random sampling of SNPs in the low-density panels. EquiLD = equidistant sampling of SNPs in the low-density panels. TOP-LD = top SNP low-density panels. Mean of accuracy ± sd across all 100 values. PBLUP = pedigree-based BLUP. HD-GBLUP = genomic based BLUP obtained with the high-density (HD) panel

**Additional file 2**: **Figure S1.** Accuracy of genomic prediction for resistance to *F. columnare* in rainbow trout, obtained with SNP panels of different densities, before and after imputation and before or after re-setting genotype missing in the HD-panel as missing after imputation. The red dotted line is the average accuracy for the HD-GBLUP (28K) prediction (0.68), the blue dotted line is the average accuracy for the pedigree-based BLUP prediction (0.59). The LD panels were created with random SNP sampling (RandLD). The blue line is the accuracy value obtained with the LD-panels (RandLD) and the dark blue line is the accuracy value obtained after imputation for those panels. The orange line is the accuracy obtained after imputation of those panels and after re-setting all the missing genotype from the HD-panel as missing in the imputed-LD-panels.

## Data Availability

The dataset supporting the conclusions of this article is available in the figshare repository, https://doi.org/10.6084/m9.figshare.21814602.v1.
