# Supplementary figures and images for: Potential of low-density genotype imputation for cost-efficient genomic selection for resistance to Flavobacterium columnare in rainbow trout (Oncorhynchus mykiss)
Source: Genet Sel Evol. 2023 Aug 14;55:59. doi: 10.1186/s12711-023-00832-z (PMC10424455; doi:10.1186/s12711-023-00832-z)

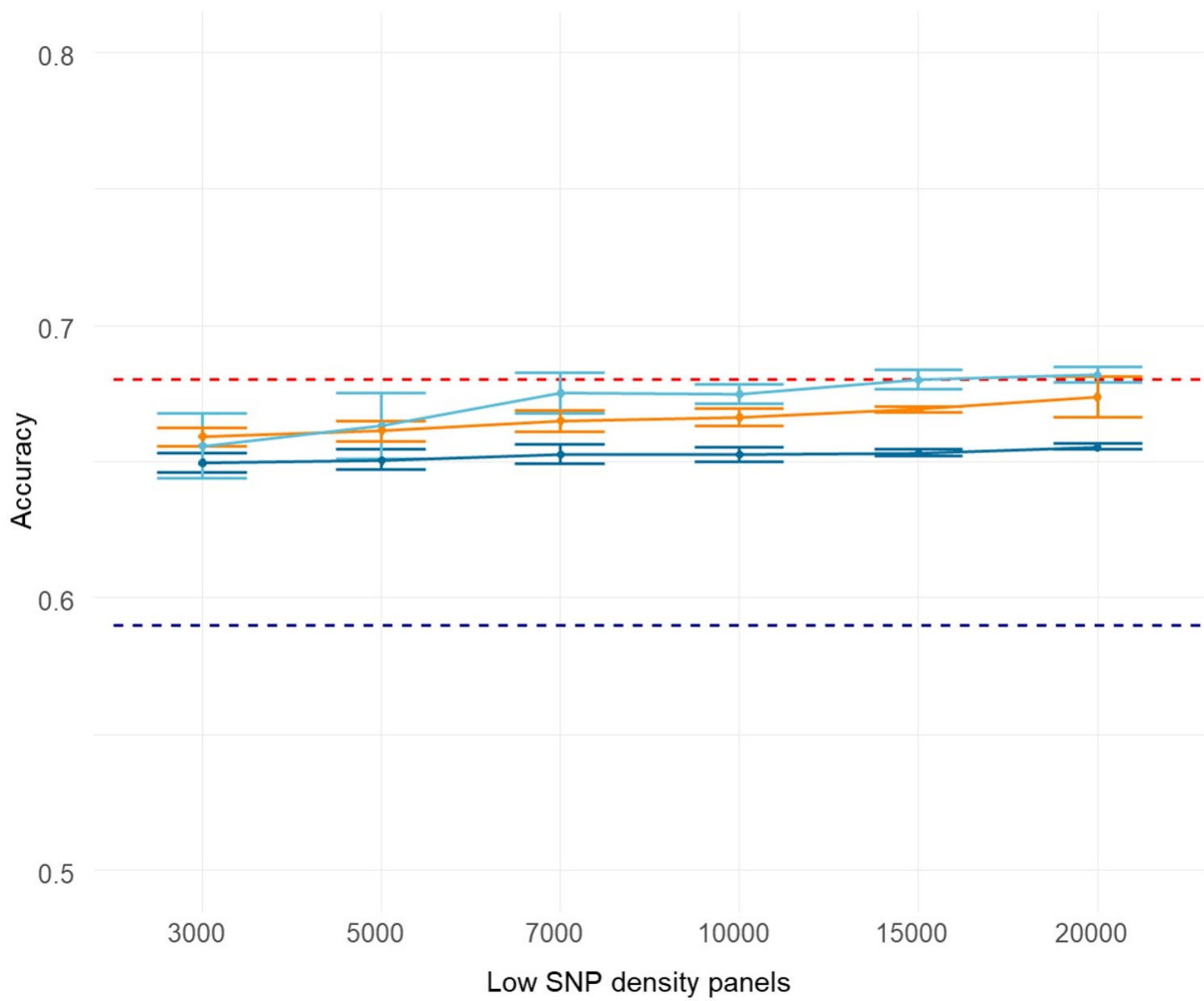

Supplement: Supplementary file 2 — Additional file 2: Figure S1. Accuracy of genomic prediction for resistance to F. columnare in rainbow trout, obtained with SNP panels of different densities, before and after imputation and before or after re-setting genotype missing in the HD-panel as missing after imputation. The red dotted line is the average accuracy for the HD-GBLUP (28K) prediction (0.68), the blue dotted line is the average accuracy for the pedigree-based BLUP prediction (0.59). The LD panels were created with random SNP sampling (RandLD). The blue line is the accuracy value obtained with the LD-panels (RandLD) and the dark blue line is the accuracy value obtained after imputation for those panels. The orange line is the accuracy obtained after imputation of those panels and after re-setting all the missing genotype from the HD-panel as missing in the imputed-LD-panels. [file 12711_2023_832_MOESM2_ESM.pdf]
